# Incidentally Visualization of the Thymus on Whole-Body Iodine Scintigraphy: Report of 2 Cases and Review of the Latest Insights

**DOI:** 10.1097/MD.0000000000001015

**Published:** 2015-07-02

**Authors:** Mahdi Haghighatafshar, Farinaz Farhoudi

**Affiliations:** From the Nuclear Medicine and Molecular Imaging Research Center (MH, FF), Shiraz University of Medical Sciences, Shiraz, Iran.

## Abstract

Radioiodine uptake is not commonly seen by the thymus gland. On the contrary, the gland is slowly replaced by fat after puberty. Herein, we present 2 patients with papillary thyroid carcinoma, follicular variant, and cervical lymph node involvement. After total/near-total thyroidectomy, the patients received ^131^I for ablation therapy. On posttreatment radioiodine scintigraphy, mediastinal ^131^I uptake was noted that finally was histologically/anatomically diagnosed as thymus gland uptake. It should be borne in mind as a potential cause of false-positive whole-body ^131^I scintigraphy.

## INTRODUCTION

Differentiated thyroid cancer (DTC) is an unusual tumor with a good prognosis when adequately treated, but can recur locally or with distant metastases even decades after initial therapy.^1^ The mainstay of treatment is total or near-total thyroidectomy. Radioiodine (^131^I) therapy is often used as an adjunct to surgery because it has been reported to improve survival rate.^2,3^ The whole body scan (WBS) with ^123^I or ^131^I is the gold standard imaging for the detection of thyroid remnant and regional or distant metastases, thus being routinely used for evaluation before and after radioiodine therapy.^4–7^ However, false-positive WBS may occur because of radioiodine uptake by normal or pathological tissues that are not related to the thyroid tissue. This could lead to unsuitable therapy, such as unnecessary surgery or administration of ^131^I. There are many normal tissues that commonly accumulate iodine, such as breasts, salivary glands, gastrointestinal, and urinary systems.^4,5^ In addition, pathological uptake has been reported in neoplastic diseases or pulmonary inflammatory, sinusitis, pericardial effusions, esophagus pathologies, urinary tract diseases, ovarian cysts or tumors, and traumatic lesions.^5–8^ Radioiodine uptake by normal thymus is an uncommon reason of false-positive WBS that might be considered as lung or mediastinal nodes metastases.^4,9–12^ It should be borne in mind to recognize this condition in order to facilitate distinction between this normal variant and metastases. We present two patients with DTC, who underwent total thyroidectomy and ^131^I therapy that had ^131^I concentrate in the mediastinum during the follow-up, and provide a review of the latest insights.

## CASE 1

**FIGURE 1:**
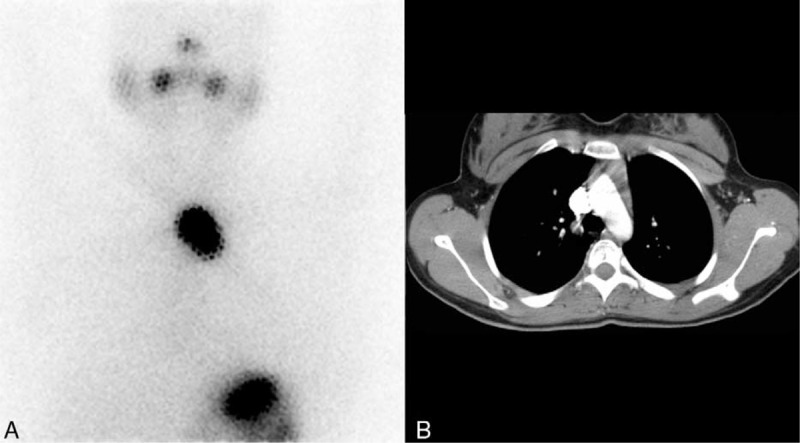
(A) Upper mediastinal uptake with no thyroid activity flowing first treatment is noted. (B) Soft tissue attenuation mass reveals in the upper mediastinum on CT scan, with histopathologic verification of thymic tissue.

A 33-year-old woman underwent total thyroidectomy and bilateral neck dissection with histopathological diagnosis of papillary thyroid carcinoma, follicular variant, and cervical lymph node involvement. She received 5550 MBq (150 mCi) of ^131^I for ablation (first ^131^I therapy). First posttreatment scan was only positive in the superior mediastinum with no evidence of cervical region uptake (Figure 1A). A computed tomography (CT) scan showed soft tissue attenuation in the mediastinum with possibility of lymph node metastasis (Figure 1B). Surgical excision was performed that subsequently showed thymic tissue.

## CASE 2

**FIGURE 2:**
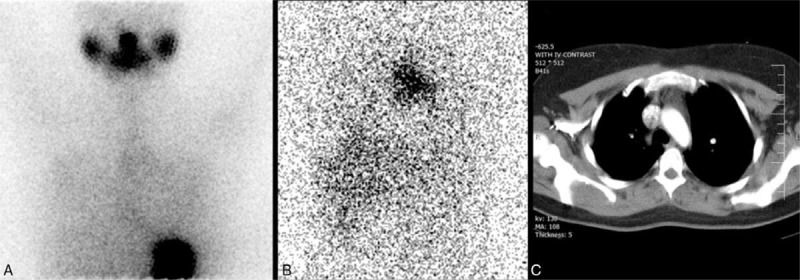
(A) Iodine-131 WBS 48 hours after second treatment reveals faint uptake in the anterior mediastinum. (B) ^131^I WBS 14 days later reveals intense uptake in the same region. (C) Soft tissue attenuation mass(thymus) reveals in the upper mediastinum on CT scan.

A14-year-old girl presented with a history of papillary thyroid carcinoma for which she underwent near-total thyroidectomy with left-side lymph node dissection and received ablative dose of 4070 MBq (110 mCi) of ^131^I 1 year ago. In first posttreatment WBS, thyroid remnant tissue with no evidence of regional or distant metastasis was noted. Diagnostic WBS was performed 12 months after ablative therapy and was in favor of lymph node metastasis in the left-side of the neck. The patient submitted to neck dissection and then received 4440 MBq (120 mCi) of ^131^I. Second posttreatment WBS was only positive in the superior mediastinum with no evidence of cervical region uptake and the uptake in the mediastinum appeared to increase from 2 to 14 days (Figure 2A, B). A CT scan showed the presence of a heterogeneous mass, bell shaped in the mediastinum, suggestive of thymus (Figure 2C), with no evidence of metastatic disease. Because the scans of the patients were anonymous and no experimental intervention was done for the patients, ethical approval was not necessary.

## DISCUSSION

Radioiodine is very valuable for the diagnosis and treatment of both benign and malignant residual thyroid tissue in patients who have submitted to thyroidectomy for DTC.^2^ Cognizance of false-positive findings in the diagnostic or posttreatment WBS in patients with DTC is essential to avoid unnecessary treatment. False-positive findings in the chest may be because of inflammatory processes, ectopic thyroid tissue, neoplasms of nonthyroidal origin, body secretions, and pathological transudates.^13^ Thymic uptake of radioiodine is one of the possible causes that can be either because of the misinterpretation of metastatic mediastinal spread or because of an intrathymic ectopic thyroid tissue,^4,9–13^ thyroidal metastases to the thymus,^14^ residual normal thymus,^2,11,12^ or thymic hyperplasia.^6,9–11^ The mechanism of iodine uptake by the thymus is not yet fully understood. Vermiglio et al^15^ considered the possibility of iodine accumulation in the Hassal's bodies present in the thymic tissue because of the similarity to the follicular cells of the thyroid. Spitzweg et al^16^ showed the presence of the human Na^+^/I symporter (hNIS) in extra thyroidal tissue, including the thymus gland, with diminished capacity to transport and concentrate iodine, compared with the thyroid gland, which could explain why higher activities, late scanning, after the ablation of the thyroid remnant, and absence of metastatic spread (loss of tissue with higher hNIS expression than the thymus), increase the possibility of visualization of the thymic tissue after administration of radioiodine.^9,13^ In the present case report, two patients presented thymic uptake in the posttherapy WBS. Surgical removal of mediastinal mass in 1 patient was done with histopathologic verification of thymic tissue, without any evidence of thyroidal or neoplastic cells. Another patient, without histological confirmation, had a CT scan indicative of thymus tissue and is in periodic monitoring. The association between thymic hyperplasia and benign thyroidal disease, especially Graves disease, is well known. The identity of Thyroid Stimulating Hormone (TSH) receptors in the human thymic tissue has also been reported.^17–20^ Niendorf et al^21^ for the first time showed relationship between thymic hyperplasia and DTC. The mechanism of this association is not thought to be similar to the thymic hyperplasia demonstrated after chemotherapy or after stress conditions or acute infections, when hyperplasia happen because of an immunological reaction.^22^ It seems that l-thyroxin hormone therapy in supraphysiological doses is responsible for the thymic hyperplasia in patients with thyroid carcinoma.^21^ Heath et al,^23^ with the technique of reverse transcription and polymerase chain reaction from analyses of mRNA, showed the thymic expression of thyroglobulin (Tg) in embryonic mice and adult rats. Sospedra et al^24^ reported transcription of Tg in 4 of 12 specimens of human thymus, and Gotter et al^25^ found the existence of Tg expression in medullary epithelial cells of human thymus. Mello et al^4^ considered the possibility of the thymic tissue as a source of benign production of Tg, and suggested randomized studies to evaluate whether the Tg generation by the thymus is actually efficient and whether it could be stimulated by TSH. Michigishi et al^9^ considered reliable criteria to distinguish malignant from benign mediastinal uptake. Thymic uptake that visualized only by higher activities on posttreatment scintigraphy becomes more intense with repeated ^131^I treatment, low serum Tg levels, younger age (<45 years) and a hyperplastic thymus indicative of nonmetastatic radioiodine accumulation. It should be born in mind that beside thymic uptake, radioiodine accumulation in the midline of the thorax may be because of various causes, for example tracer accumulation in the esophagus, hiatal hernia, and aspiration of radioactive saliva in the tracheobronchial tree.^13,26^
